# Supplementary material for: Simulating the next steps in badger control for bovine tuberculosis in England
Source: PLoS One. 2021 Mar 18;16(3):e0248426. doi: 10.1371/journal.pone.0248426 (PMC7971561; doi:10.1371/journal.pone.0248426)
Supplement: S1 Appendix — (DOC) [file pone.0248426.s001.doc]

# S1 Appendix. Model Variables (Temporal Settings)

| Year badger population initialised | 1990 |
| --- | --- |
| Years of badger control (intensive culling) | 2016-2019 |
| Years of badger control (exit strategy) | 2020-2050 |
| Last Year of each simulation | 2050 |
