# Supplementary material for: Simulating the next steps in badger control for bovine tuberculosis in England
Source: PLoS One. 2021 Mar 18;16(3):e0248426. doi: 10.1371/journal.pone.0248426 (PMC7971561; doi:10.1371/journal.pone.0248426)
Supplement: S2 Appendix — (DOC) [file pone.0248426.s002.doc]

# S2 Appendix. Model Variables (Spatial Settings)

| Total area simulated (km^2^) | 1600 |
| --- | --- |
| Control area (km^2^) | 400 |
| Model grid resolution | 200 x 200 cells |
| Number of badger social groups | 1200 |
| Mean badger territory area (km^2^) | 1.33 |
