# Supplementary material for: Simulating the next steps in badger control for bovine tuberculosis in England
Source: PLoS One. 2021 Mar 18;16(3):e0248426. doi: 10.1371/journal.pone.0248426 (PMC7971561; doi:10.1371/journal.pone.0248426)
Supplement: S3 Appendix — (DOC) [file pone.0248426.s003.doc]

# S3 Appendix. Model Variables (Badger and Cattle Parameters)

**Appendix 3.1 Badger s**ettings:

| **Initial badgers added to social group**  Figures obtained by iterative process to give ratio of badger ages and sexes. | **England** |
| --- | --- |
| Juvenile male | 0 or 1 (mean 0.8) |
| Yearling male | 0 or 1 (mean 0.6) |
| Adult male | 1 or 2 (mean 1.2) |
| Juvenile female | 0 or 1 (mean 0.9) |
| Yearling female | 0 or 1 (mean 0.7) |
| Adult female | 2 or 3 (mean 2.1) |

| **Mortality rates** Note: these probabilities are adjusted to be linearly inversely proportional to group size so smaller groups have lower mortality rates. The adjustment uses a mortality rate multiplication factor based on the equation: 1 – (0.07 * ([SocialGroupAverage] – [GroupSize])), where SocialGroupAverage is set to 5.7. 6.7. or 7.5 depending on whether the social group has a carrying capacity of 2, 3, or 4 respectively. | |
| --- | --- |
| male 1st 2m pre-emergence | 0.2400 |
| female 1st 2m pre-emergence | 0.2400 |
| male ELISA negative (healthy) | 0.0637 |
| Female ELISA negative (healthy) | 0.0500 |
| Male ELISA positive | 0.0908 |
| Female ELISA positive | 0.0513 |
| Male single site excretor | 0.1168 |
| Female single site excretor | 0.0479 |
| Male multi-site excretor | 0.2831 |
| Female multi-site excretor | 0.1461 |
| **Breeding probabilities** |  |
| First female | 0.85 |
| 2nd female [adjustable^(1)^] | 0.40 +/- |
| 3rd female [adjustable^(1)^] | 0.40 +/- |
| 4th female [adjustable^(1)^] | 0.40 +/- |
| ^(1)^ Note: the probabilities of 2^nd^/3^rd^/4^th^ female breeding are adjusted to be linearly inversely proportional to group size – so smaller groups may breed back up to size faster. The adjustment is based on the equation: 0.40 + ([GroupSize] - 6.7) * -0.079, but limited between the values 0.00 and 0.85. | |
| **Litter size probabilities**  Taken from (p160) |  |
| 1 cub | 0.08 |
| 2 cubs | 0.18 |
| 3 cubs | 0.51 |
| 4 cubs | 0.18 |
| 5 cubs | 0.05 |
| **Dispersal probabilities** |  |
| Male | 0.009390 |
| Female | 0.000834 |
| **Health-status transfer probabilities**  Taken from |  |
| male ELISA positive to single site excretor | 0.0479 |
| female ELISA positive to single site excretor | 0.0316 |
| male ELISA positive to multi-site excretor | 0.0140 |
| female ELISA positive to multi-site excretor | 0.0094 |
| male single site excretor to multi-site excretor | 0.0727 |
| Female single site excretor to multi-site excretor | 0.0479 |

| **Infection transmission probabilities**  Rates set to give a badger prevalence and CHB rate appropriate for each region | **England** |
| --- | --- |
| single site excretors |  |
| badger to badger within group | 0.036 |
| badger to badger between group | 0.0018 |
| multi-site excretors |  |
| badger to badger within group | 0.072 |
| badger to badger between group | 0.0036 |

| **TB-test probabilities** |  |
| --- | --- |
| TB-Test sensitivity (ELISA positive badger positive) | 34% |
| TB-Test sensitivity (single site excretor badger positive) | 42% |
| TB-Test sensitivity (multi-site excretor badger positive) | 78% |
| TB-Test specificity (healthy badger negative) | 95% |

**References**

1. Graham J, Smith G, Delahay R, Bailey T, McDonald R, Hodgson D. Multi-state modelling reveals sex-dependent transmission, progression and severity of tuberculosis in wild badgers. Epidemiology and infection. 2013;141(07):1429-36.

2. Neal E, Cheeseman C. Badgers. London: T & AD Poyser ltd; 1996.
