# Supplementary material for: Simulating the next steps in badger control for bovine tuberculosis in England
Source: PLoS One. 2021 Mar 18;16(3):e0248426. doi: 10.1371/journal.pone.0248426 (PMC7971561; doi:10.1371/journal.pone.0248426)
Supplement: S4 Appendix — (DOC) [file pone.0248426.s004.doc]

**S4 Appendix. Model Processes (submodels)**

1. *Model scheduling*
   Within each two-month time step the following sequential processes occurred:
2. Reproduction (time-step 1 only = February)
3. Ageing (time-step 1 only = February)
4. Mortality
5. Dispersal
6. Badger Control
7. Social Perturbation
8. Disease transmission
9. Disease progression
10. Data output

The above processes (sub-models) are described in more detail elsewhere , along with a description of the processes allowing population and disease dynamics to stabilize before badger control is simulated.

1. *Creation of badger territories*
   A specified number of badger territories are created: main setts are placed randomly across the grid, and grid squares allocated to closest main sett to give fully contiguous tessellated badger groups. The grid is treated as a torus so there are no edges. The total number of badger groups added to the grid (see Appendix S2) gives an average territory size of 1.33km^2^ and mean territory densitiy of 0.75km^-2^.
2. *Creation of Farms*A specified number of farms are added to the grid to produce a realistic farm density of 0.78 km^-2^. Farm centres are added to the grid at random and farm land is contiguously tesselated around the centre points. The participation of farms within the control zone is randomly allocated to give an overall participation rate of 70%. The spatial distribution of farms is used to define the border of the badger control zone as it would be in practice.
3. *Definition of neighbours*This procedure determines the neighbours of each social group, allowing between-group TB transmission to be simulated.
4. *Addition of badgers*Badgers are added to each badger territory at the start of year one (1990), to give a stable mean badger group size of about 6.7 adult badgers per group, as measured at the end of December.
5. *Reproduction*In the model female badgers give birth in the first time step of each year, which is equivalent to January + February. The number of females that breed in any one social group is determined probabilistically (Appendix S3), although this is limited by the number of 2+ yr-old females, and the carrying capacity of the group. The breeding probability for the first female is fixed, but the probabilities of the 2nd/3rd/4th are higher for groups with fewer badgers present (linear relationship). Litter sizes are also determined probabilistically, mean litter size is 2.94, and the cub male : female ratio is 1:1.
6. *Ageing*
   This occurs in the first time step of each year within the “birth of badgers” procedure. All badgers are aged by one year immediately after the birth routine, just before the new cubs are added to the main population array.
7. *Mortality*Badger mortality rates , are dependent on sex, age, and health status (Appendix S3), and are adjusted linearly to give lower mortality rates for smaller groups. The mortality rates are applied to individual badgers probabilistically.
8. *Dispersal*Dispersal probabilities are sex-dependent (Appendix S3), but are not related to age or season. The dispersal routine occurs every time-step. Badgers disperse only as far as their neighbouring group, and tend to move to a group with fewer badgers if one is available. Badgers are not allowed to disperse twice in one time-step.
9. *Social perturbation of badgers*This procedure moves badgers to fill vacancies. It occurs whether or not control is being simulated, but obviously these perturbation movements are more frequent immediately following badger removal. Sexes are checked independently; groups that already have two of a sex would not receive a third, and the donor group must also have at least three more badgers of that sex than the recipient group. Badgers are moved shorter distances in preference, and a badger is not allowed to make two moves within the same time-step.
10. *Seeding of TB in badgers*At the start of year 5 (1995), each badger group is given a high probability to have one badger of random sex and age to be infected with TB. Each selected badger during this seeding process is given a TB status of “ELISA positive”.
11. *Transmission of TB – badger to badger*Each infectious badger has a chance of infecting every contact, both within-group, and between-group (neighbours). Transmission rates are set higher for multi-site excretor badgers, and between-group rates are set to 5% of within group rates (Appendix S3 and ). During years of badger control, between-group infection rates are recalculated (see “*Setting Transmission Rates*” below) to give higher TB transmission rates in/around the control area (i.e. a higher probability of between-group contacts). This is to simulate the “perturbation effect” of badger culling.
12. *Setting transmission rates (including perturbation effect)*Transmission rates are applied stochastically, and default values are listed in Appendix S3. Special rates, however, are applied to those badger groups subjected to culling, and to their immediate neighbors. This is to simulate higher contact rates during a period of social perturbation as a result of the culling. This perturbation effect lasts one year following culling so is in effect from the first cull to one year after the last cull in the intensive culling period. Wherever and whenever the perturbation effect is applied, all badger-to-badger between-group transmission rates are increased to equal the within-group rates.
13. *Disease progression*Badgers with TB are given the chance of transferring from one TB-status to another, according to set probabilities (Appendix S3). A badger can only make one such change per time step. Disease progression is from ELISA positive to single site excretor to multi-site excretor and all changes are unidirectional. A newly ELISA positive badger does not itself have the chance to infect another badger until the following time-step.
14. *Apply badger control*Each control method is applied for from 2020 to 2050 inclusive following the period of intensive culling. Each method simulated starts with identical conditions at the start of year 2020, to give a fair comparison between the control methods. The badger groups to be controlled are determined by their location within the central core zone. A proportion of farms in the core are excluded at random from having badger control, to simulate non-compliance. Control of the selected badger groups is applied stochastically at the specified control rate for the method.
15. *Save data*
    Output parameters are calculated at the same point each year, including badger population, badger TB (number and prevalence) and number controlled. All output data for each set of simulations is saved in one Excel file.

**References**

1. Wilkinson D, Bennett R, McFarlane I, Rushton S, Shirley M, Smith G. Cost-benefit analysis model of badger (Meles meles) culling to reduce cattle herd tuberculosis breakdowns in Britain, with particular reference to badger perturbation. Journal of Wildlife Diseases. 2009;45(4):1062-88.

2. Graham J, Smith G, Delahay R, Bailey T, McDonald R, Hodgson D. Multi-state modelling reveals sex-dependent transmission, progression and severity of tuberculosis in wild badgers. Epidemiology and infection. 2013;141(07):1429-36.

3. Smith GC, Cheeseman CL, Wilkinson D, Clifton-Hadley RS. A model of bovine tuberculosis in the badger *Meles meles*: the inclusion of cattle and the use of a live test. J Appl Ecol. 2001;38:520-35. doi: 10.1046/j.1365-2664.2001.00610.x.
