# Supplementary material for: Simulating the next steps in badger control for bovine tuberculosis in England
Source: PLoS One. 2021 Mar 18;16(3):e0248426. doi: 10.1371/journal.pone.0248426 (PMC7971561; doi:10.1371/journal.pone.0248426)
Supplement: Attachment — Submitted filename: Godfray exit Response to referees.docx [file pone.0248426.s005.docx]

Response to referees

**Comments to the Author**

Reviewer #1: This useful and timely analysis compares different models of controlling badger populations, to reduce risk of BTB spread to cattle. The main claims of the paper are that the 3 control approaches are projected to result in similar reduction in infected badgers, based on statistical modeling.
The data and analyses support the claims.
However, the manuscript could be revised to make it clear to non-specialists in the field.
For example, on page 2 authors could elaborate further on reference 10 about the desirability of non-lethal control models. BCG vaccination protocols could be briefly explained as 2 options (injection versus oral baiting) could be utilized.

There is no oral vaccine available, nor under investigation in the UK. I have clarified that “vaccination, and fertility control, was performed on trapped animals” in lines 47 and 52-54
The Discussion ends without a summary of conclusions, which are found only in the Abstract. Assuming the 3 control methods are equivalent, then the authors should advance how wildlife managers would make a selection, i.e. cost analyses as well.

This is a very good point, and I have added some test in the early discussion to clarify the accuracy of the simulation, plus a summary at the end of the discussion about preference between strategies and the relative costs.

Reviewer #2: The modeling work presented in the paper addresses the important question of the benefit of deploying vaccines to control TB in badgers and cattle in the UK, under the current situation of industry-deployed cull.
The vaccine considered for the model is injectable (by IM delivery), which should be mentioned (for ex on L103 and L157) since it implies the need for trapping badgers, contrarily to oral vaccination which could be more widely deployed and potentially reach a larger range of animals/groups.

There is no oral vaccine available, nor under investigation in the UK. I have clarified that “vaccination, and fertility control, was performed on trapped animals” in lines 52-54
The model is based on the TB situation in the UK, which should be specified more in the paper, for example l41 “it is not certain what level of effect this would have on bTB incidence in cattle in the UK”.

I’m not sure how to specify this more clearly. It is in the title, but I have added “in England” at lines 21 and 48 to ensure the reader is aware this simulation is after the current English badger culling policy has expired.
The output of number of infected badgers only is surprising, especially as the author mentions in L173-174 that prevalence is the measure most likely to be available in the field. The author may be more explicit on the rational for choosing this output, possibly that the risk to cattle may be expected to be proportional to the number of infected animals, not to their density, and this should be discussed.

Text changed
Finally, the overall conclusion could be clearer. Is vaccination a favorable strategy compared with culling?

See additional text at the end of the discussion (second point above)
Corrections and new formulations are suggested below:
L84: Justify why the prevalence of bTB in badger population was fixed at 14%

Text amended to include a reference.
L108, is the fertility control treatment vaccination given yearly in the animals trapped for injectable vaccination, as suggested in the legend of fig1?

Text changed to confirm this

Rephrase L173-174.

Text changed
L170: replace reduce by decrease

Text changed
L175: add “in badgers” after “infection”

Text changed
L176: add “bTB” before eradication

Text changed
Rephrase L179-180.

I have edited this to two sentence to make the text clearer.
Rephrase L184 from “so reinvasion was slow”.

Reworded
L184, be more specific about the strategy with additional benefit. The author should also mention the lack of immigration in the context of vaccination, given the return of the population number to social equilibrium between the groups.

Additional text added at line 194 to clarify this.
L187 The author mentions the (non existant) welfare advantage of culling strategies but not of vaccination exit strategy involving vaccination.

Text inserted at the start of this paragraph
L192, add” than for culling” after “was much higher”

Text changed
L193, Replace “whereas” by “when”, rephrase after “this resulted”

Text adjusted
L195, add “with BCG” after vaccination and “individual” before protection, and “vaccinated” before animals

Changed
L198, be more precise in term of other work in hand to examine the overall efficacy of vaccination

I have clarified this point in the text.
